# TNFα/IFNγ Mediated Intestinal Epithelial Barrier Dysfunction Is Attenuated by MicroRNA-93 Downregulation of PTK6 in Mouse Colonic Epithelial Cells

**DOI:** 10.1371/journal.pone.0154351

**Published:** 2016-04-27

**Authors:** Ricci J. Haines, Richard S. Beard, Rebecca A. Eitner, Liwei Chen, Mack H. Wu

**Affiliations:** 1 James A. Haley Veterans’ Hospital, Tampa, Florida, United States of America; 2 University of South Florida, Morsani College of Medicine, Department of Surgery, Tampa, Florida, United States of America; 3 University of South Florida, Morsani College of Medicine, Department of Molecular Pharmacology and Physiology, Tampa, Florida, United States of America; Duke University Medical Center, UNITED STATES

## Abstract

Since inflammatory bowel diseases (IBD) represent significant morbidity and mortality in the US, the need for defining novel drug targets and inflammatory mechanisms would be of considerable benefit. Although protein tyrosine kinase 6 (PTK6, also known as breast tumor kinase BRK) has been primarily studied in an oncogenic context, it was noted that PTK6 null mice exhibited significantly enhanced colonic epithelial barrier function. Considering that the inflammatory functions of PTK6 have not yet been explored, we hypothesized that cytokines responsible for mediating IBD, such as TNFα/IFNγ, may solicit the action of PTK6 to alter barrier function. After first assessing critical mediators of TNFα/IFNγ driven epithelial barrier dysfunction, we further explored the possibility of PTK6 in this inflammatory context. In this report, we showed that PTK6 siRNA and PTK6 null young adult mouse colonic epithelial cells (YAMC) exhibited significant attenuation of TNFα/IFNγ induced barrier dysfunction as measured by electric cell-substrate impedance sensing (ECIS) assay and permeability assays. In addition, PTK6 null cells transfected with PTK6 cDNA displayed restored barrier dysfunction in response to TNFα/IFNγ, while the cells transfected with vector alone showed similar attenuation of barrier dysfunction. Furthermore, using subcellular fractionation and immunocytochemistry experiments, we found that PTK6 plays a role in FoxO1 nuclear accumulation leading to down-regulation of claudin-3, a tight junction protein. Moreover, we searched for relevant miRNA candidates putative for targeting PTK6 in order to identify and assess the impact of microRNA that target PTK6 with respect to TNFα/IFNγ induced barrier dysfunction. Subsequently, we assayed likely targets and determined their effectiveness in attenuating PTK6 expression as well as cytokine induced barrier dysfunction. Results showed that miR-93 reduced PTK6 expression and attenuated TNFα/IFNγ imposed decrease in transepithelial electrical resistance (TER), as well as excluded FoxO1 from the nucleus. Our results indicate that PTK6 may act as a novel mediator of intestinal epithelial permeability during inflammatory injury, and miR-93 may protect intestinal epithelial barrier function, at least in part, by targeting PTK6.

## Introduction

Abnormal intestinal epithelial barrier function is often observed in patients with inflammatory bowel diseases characterized by inflammation driven relapsing diarrhea [1, 2]. The increase in paracellular permeability resulting from inflammation promotes antigen exposure to underlying immune cells, thereby enhancing intestinal inflammation. TNFα and IFNγ are proinflammatory cytokines involved in mediating the intestinal epithelial barrier dysfunction observed in inflammatory bowel diseases, and numerous studies have demonstrated their role in disruption of epithelial apical junction structure [3–5]. Importantly, the signaling events contributing to epithelial barrier dysfunction observed by TNFα and IFNγ are largely due to apoptotic-independent mechanisms such as activation of Src-related kinases [6].

The distantly Src-related protein kinase termed protein tyrosine kinase 6 (PTK6) has been identified as playing a role in intestinal epithelial barrier function in that PTK6 knockout led to several fold increase in basal intestinal epithelial barrier function as evidenced by resistance measurements of cultured monolayers [7, 8]. In addition, it has been shown that several key components of the cell-cell junction and cell-matrix adhesions are directly phosphorylated by PTK6, including paxillin [9], β-catenin [2], and focal adhesion kinase [10]. Other proteins that modulate barrier function are known to associate with PTK6 via its SH3 domains, including ADAM 15 [11]. Furthermore, it has been demonstrated that PTK6 directly inactivated the protein kinase Akt in a manner that led to increased nuclear accumulation of FoxO1 [12], a transcription factor known to regulate inflammatory responses in epithelial cells [13]. Importantly, it has been shown that nuclear accumulation of FoxO1 is necessary for the downregulation of tight junction proteins in response to cytokine stimulation *in vitro* [14]. Other studies have shown that FoxO1 knockdown improved ethanol induced intestinal epithelial barrier dysfunction, suggesting a potential for FoxO1 in regulating epithelial junction dynamics [15]. Taking this information together, we sought to determine whether PTK6 contributes to TNFα and IFNγ mediated epithelial barrier dysfunction in a FoxO1 dependent manner.

Barrier function is a dynamic process that demands regulation at the epigenetic level. As shown by Ghatak et al, interruption of the machinery required to process microRNA resulted in high trans-epidermal water loss in mice [16]. Furthermore, it was found that microRNAs were aberrantly regulated in inflamed mucosa of patients experiencing acute Crohn’s Disease symptoms [17]. Considering these findings, it is not surprising that microRNA have shown promise as a therapeutic strategy in several diseases where traditional means of intervention have been elusive. For example, it has been shown that the targeting of KRAS by exogenous administration of the let-7 mimic robustly inhibited tumor growth in a mouse model [18], and this strategy is in preclinical development. It is thought that identifying novel microRNA-mRNA relationships may potentially hold therapeutic value for a number of diseases, including those that are characterized by epithelial barrier dysfunction. Therefore, in addition to determining mechanism characteristics of PTK6 involvement in epithelial barrier dysfunction, we sought to identify putative microRNA regulators of PTK6 and whether improvement of epithelial barrier dysfunction can be achieved upon exogenous microRNA administration. With *in silico* modeling and bioinformatic analysis, we identified a microRNA (miR-93) as being highly likely to target PTK6 mRNA in humans and mice. MicroRNA-93 is a member of the miR-17 family and is known to play a role in inflammation and barrier function. Specifically, miR-93 expression was shown to be positively correlated with increase in epithelial barrier function [16]. Accordingly, our hypothesis was that miR-93 mediated inhibition of PTK6 expression improves inflammation driven intestinal epithelial barrier dysfunction in a manner that prevents FoxO1 nuclear accumulation. Our results suggest that miR-93 targeting of PTK6 may be barrier protective for intestinal epithelium under inflammatory conditions.

## Materials and Methods

### Cell Culture

The conditionally immortalized cell line, young adult mouse colonic epithelial cells (PTK6+/+ = “wild type” or WT YAMC; and PTK6-/- YAMC) were a gift from Dr. Whitehead at Vanderbilt University [7, 19, 20]. Briefly, colonic epithelial cells from transgenic mice expressing a temperature sensitive simian virus 40 large tumor antigen (tsA58 SV40 TAg) were isolated using standard protocols. After encouraging expression of tsA58 TAg with 5U/ml IFNγ and 33°C to demonstrate immortalized cell growth and allow cells to reach confluence, media was changed to IFNγ-free media and cells were grown at 37°C 24 hours to quench immortalized phenotype. PTK6-/- cells were isolated from transgenic mice expressing tsA58 SV40 TAg with the PTK6 gene interrupted [7, 21] and were grown under identical conditions. For miR-93, PTK6 siRNA (mouse), and PTK6 cDNA (mouse) transfecton experiments, WT YAMC were transfected with siRNA (IDT), miR-93 (Qiagen), or PTK6 cDNA (Origene) or scrambled control/ empty vector via electroporation in electroporation solution (Mirus) using a Bio-Rad Gene Pulser Xcell according to manufacturer’s instructions. Breifly, cells were trypsinized and harvested then resuspended in Mirrus solution at at 5 X 10^6^ cells/ml. Final concentrations of RNA were 300 nM per electroporation reaction and 20 μg/ml for cDNA. Cells were plated in Collagen I coated plates or transwells prior to further treatment. Cells were treated with a cytokine cocktail (TNFα [100ng/ml]/ IFNγ [500U/ml], Sigma) 24 hours (unless otherwise noted) at mortalization temperature (37°C) before analysis.

### Electric cell-substrate impedance sensing

YAMC barrier function was determined as previously described [22] by measuring cell-cell adhesive resistance to electric current using an electric cell-substrate impedance sensing (ECIS) system (Applied Biophysics, Troy, NY). Briefly, 2 x 10^5^ epithelial cells were seeded on ECIS electrode arrays pre-coated with collagen I. After adhering ~16 hours, the arrays were affixed to the ECIS system (1-V, 4000-Hz alternating current signal supplied through a 1-MΩ resistor to a constant-current source, in-phase voltage and out-of-phase voltage were recorded with ECMS 1.0 software (CET, IA, USA)). ECIS tracings expressed as transepithelial electric resistance (TER) are normalized to plateaued resistance values, and comparisons were made between TNFα/IFNγ cocktail ([100 ng/mL]/ 500U/ml) and vehicle control ([0.1% BSA in PBS]) treated YAMC monolayers. TER changes were recorded every 90 seconds. Results are representative of 3 independent experiments.

### Quantitative Real-time PCR

YAMC treated (cytokine cocktail: (TNFα [100ng/ml]/ IFNγ [500U/ml]) or vehicle control (VC) 0.1% BSA in PBS) 16 hours were lysed in RNAzol (Molecular Research Center, Inc) followed by RNA extraction as described by manufacturer. The mRNA fraction was quantified and equal masses of RNA were reverse transcribed to cDNA using random hexamers prior to qPCR. Samples without reverse transcriptase (No RT) were run as negative control. Equal volumes of cDNA were used to quantify PTK6 message using specific primers (Bio-Rad) and SYBR master mix (Bio-Rad) according to manufacturer’s instructions. Samples were assayed in triplicate and No RT controls were used to ensure absence of genomic DNA (data not shown). Relative mRNA expression levels were determined by using GAPDH as the housekeeping gene via CFX96 Touch Real-Time PCR detection system and software (Bio-Rad). Data are representative of 4 experiments *p<0.05.

### Permeability assay

Sodium fluorescein-flux assays were conducted as previously described [23]. WT or PTK6 -/- YAMC were plated on collagen I coated polycarbonate 12-well transwell inserts (0.4μM pore size, Corning) and allowed to reach confluence. Then, the luminal (top) compartment was treated with sodium fluorescein (2.5 mg/ml) for 15 minutes, then 100 μl aliquots were obtained from each abluminal (bottom) compartment and assayed for FITC fluorescence. WT YAMC samples were diluted 1/25 in order to yield fluorescence values within standard curve. Permeability coefficients (P_s_) were calculated using the formula P_s_ = [Ab]/t× 1/A×V/[Lu], where [Ab] is the abluminal concentration of sodium fluorescein, t is time in seconds for sodium fluorescein incubation, A is area of membrane in cm^2^, V is the volume of the abluminal chamber, and [Lu] is the luminal concentration of sodium fluorescein. Results are representative of 4 experiments *p<0.05.

### Immunocytochemistry

WT or PTK6-/- YAMC monolayers were grown in 2-chamber slides (Nunc) for immunocytochemistry analysis. Fixation and immunolabeling were performed using standard protocols. Briefly, cells were washed twice [PBS with 2mM CaCl2, 2mM MgCl2] followed by incubation in 4% paraformaldehyde for 10 minutes. Slides were rinsed twice in PBS, permeabilized [PBS, 0.1% Triton X-100], then blocked [PBS, 3% BSA, 0.01% Tween-20] for 2 hours at room temperature. Cells were incubated with primary antibodies (ZO-1, Cell Signaling/ FoxO1, Millipore) 16 hours at 4°C diluted 1:50 [PBS, 0.01% Tween-20, 3% BSA], washed 3 times for 10 minutes each [PBS, 0.1% Tween-20], incubated with appropriate secondary antibody diluted 1:200 [PBS, 0.01% Tween-20, 2% BSA] (AlexaFluor-conjugated; Life Technologies). Slides were mounted with anti-fade media containing DAPI (Life Technologies). Immunofluorescence confocal microscopy was performed with Olympus FV1000 MPE multiphoton laser scanning microscope (Olympus). Images were performed from 3 independent experiments and representative confocal micrograph images are presented. Images were analyzed using Image J software as previously described [24] with modifications. Briefly, for ZO-1 organization measurement, intensity at cell junctions were sampled at random followed by quantification of cytosolic intensity and a ratio was generated per treatment group as a measure of ZO-1 cytosolic translocation. Similarly, nuclear FoxO1 intensity was sampled at random and averaged among different treatment groups then compared statistically for determining significance (ANOVA followed by Student’s t-test).

### Subcellular fractionation

Treated YAMC (TNFα [100ng/ml]/ IFNγ [500U/ml]) or vehicle control (VC) 0.1% BSA in PBS 16 hours) were subjected to nuclear fractionation in order to assess changes in FoxO1 nuclear accumulation relative to treatment and PTK6 expression status. Cells were lysed in fractionation buffer (250 mM sucrose, 20 mM HEPES pH 7.4, 1.5 mM MgCl2, 10 mM KCl, 1 mM EDTA, 1 mM EGTA, 1 mM DTT, 1X phosphatase inhibitor cocktail (Pierce)) followed by several passages through 25 G needle. Nuclear pellets were obtained by centrifugation (3,000 RPM, 4°C, 5 minutes) and washing 4 X in PBS. Pellets were then sonicated and resuspended in nuclear buffer. The nuclear fractions were subjected to BCA protein assay (Pierce) prior to Western blot analysis. Equal masses of protein were loaded to 4–20% polyacrylamide gels (Bio-Rad) for SDS-PAGE followed by Western blotting using FoxO1 antibody (Cell Signaling), or lamin A/C antibody (Cell Signaling) to ensure equal protein loading of nuclear proteins. Results are representative of 3 individual experiments.

### Bioinformatic analysis

Identifying microRNAs for study was performed by analyzing the PTK6 3’UTR at several levels (S2 Fig). First, predicted miRNA were identified using MirWalk “Gene-miRNA” prediction algorithm. This analysis produced 140 unique miRNA sequences. From this list, five microRNA known to be involved in inflammation or barrier function were identified for further analysis (S2 Fig). Probability scores on these five sequences were considered a “hit” when p<0.05 using MiRanda, PicTar, RNA22, and TargetScan. Next, a pairwise alignment of the PTK6 3’UTR for human and mouse was conducted to determine areas of conservation. The reverse complement of the seed sequence was searched for in the human or mouse PTK6 3’UTR (Ensemble) as well as in the pairwise alignment. Two sequences showed binding potential in areas conserved between mice and humans (miR-518 and miR-93). Mir-93 scored the highest in all categories.

### Luciferase assays

The confirmation of miR-93 targeting the 3’UTR of PTK6 was determined by using the LightSwitch Luciferase assay system (SwitchGear Genomics). YAMC cells were plated at 5 X 10^5^ cells/ml in a white 96 well culture plate the day before initiating the assay. On the following day, 30ng/μl of the PTK6 3’UTR, mutated PTK6 3’UTR (mutated at putatitive miR-93 binding sites), or reporter vector only were co-transfected with 100nM miR-93, mutated miR-93, scrambled sequence, or miR-518 for 24 hours. The luciferase assay was performed the following day according to the manufacturer’s instructions. Knockdown was calculated by determining the luciferase signal ratio for each specific combination and comparing that to signal intensity for the reporter vector and scrambled sequence. Experiments were performed in triplicate and repeated three times for statistical significance.

### Statistics

One-way analysis of variance was used to determine significantly different changes among sets of 3 or more groups. Significance (p<0.05) was noted between treated and vehicle control groups using Students *t*-test. Results are representative of 3 independent experiments unless otherwise stated.

## Results

### The cytokine cocktail TNFα and IFNγ induced barrier dysfunction is mediated by PTK6 upregulation in YAMC

**Fig 1:**
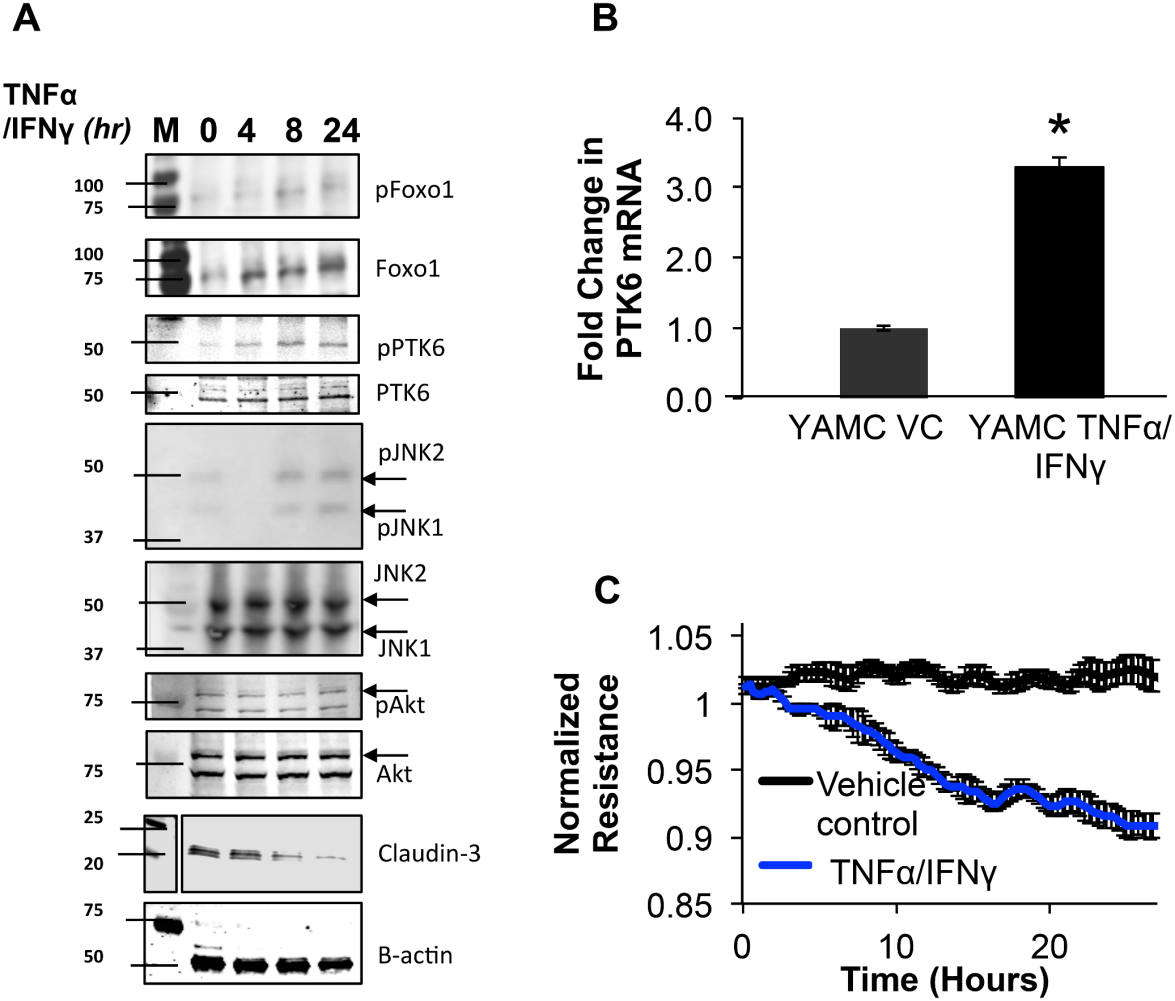
TNFα/IFNγ mediated barrier dysfunction involves activation of PTK6, JNK, and downregulation of claudin-3 in YAMC. A) Mortalized, 2-day post-confluent YAMC monolayers were treated with vehicle control (0.1% BSA in PBS) or TNFα (100ng/ml) and IFNγ (500U/ml) at the indicated time points then assayed for changes in expression of the indicated proteins (please refer to S3 Fig for full blot scans). B) RNA was isolated then quantitated for PTK6 levels with qPCR. Error bars represent standard error for 3 separate experiments (*p<0.05, n = 3). C) YAMC were added at 2 X 10^5^ cells/ml to ECIS arrays (8W10E+), allowed to reach 2-day post-confluence, mortalized, then treated with either vehicle control (VC) or TNFα (100ng/ml) and IFNγ (500U/ml). Resistance measurements were recorded every 90 seconds and values were normalized to timepoint zero. Shaded area represents standard error (n = 4).

Since colonic epithelial cells from PTK6-/- mice showed enhanced barrier function under basal conditions, we wanted to determine if the expression of PTK6 was regulated by inflammatory stimulation. Therefore, WT YAMC were grown to confluence then treated with TNFα/IFNγ cocktail or vehicle control (VC) 16 hours followed Western blotting and qPCR. In order to examine the precise role that PTK6 may play in YAMC under these conditions, we probed for a number of signaling molecules known to mediate pathways that characterize epithelial inflammation. Results showed that TNFα/IFNγ (100ng/ml, 500U/ml) induced PTK6 expression at the level of mRNA and phosphorylation at Y342 (Fig 1A and 1B), suggesting that stimulation with these cytokines regulated PTK6 expression at the level of mRNA and post-translationally in YAMC. In addition, increased phosphorylation JNK (pT183/pY185), and FoxO1 (pS256) was observed with respect to time. Phosphorylation of Akt (pT473) showed a slight decrease at the eight-hour time point, but these results were not statistically significant. The tight-junction protein claudin-3 showed significant downregulation with respect to time. Electric cell-substrate impedance sensing with YAMC monolayers confirmed that YAMC respond to TNFα/IFNγ similarly to other cell models (Fig 1C).

**Fig 2:**
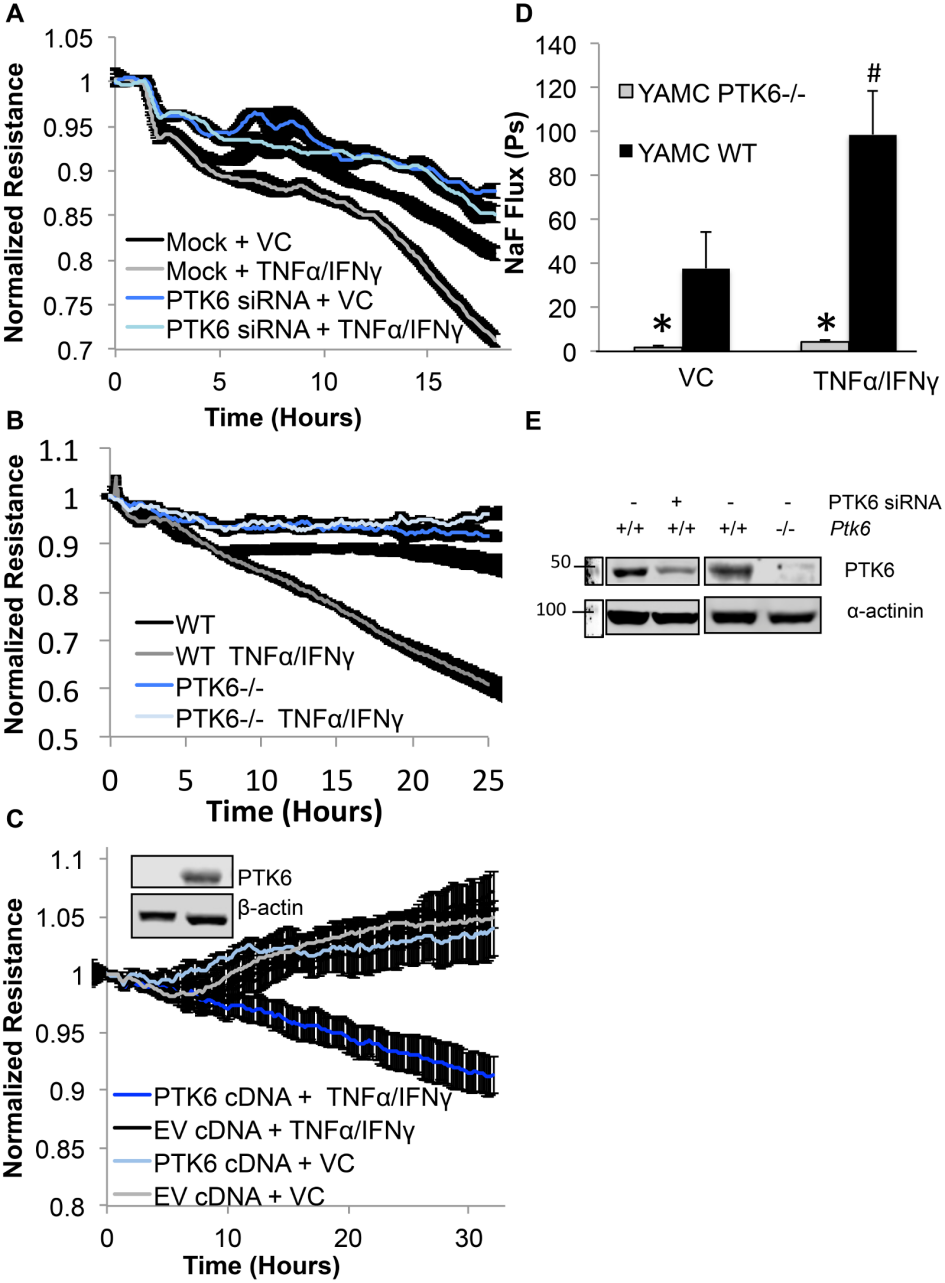
Targeting PTK6 expression improves TNFα/IFNγ mediated barrier dysfunction. A) YAMC were electroporated with PTK6 siRNA or vehicle control (TE) then plated on ECIS arrays and allowed to express for 48 hours. Monolayers were then treated with vehicle control or indicated cytokines then resistance measurements were recorded every 90 seconds B) Either WT or PTK6-/- YAMCs were grown on gold-plated ECIS arrays, stimulated with either vehicle control (VC) or TNFα (100ng/ml) and IFNγ (500U/ml), then resistance measurements were recorded every 90 seconds. Values were normalized to timepoint zero and shaded area represents standard error (n = 4). C) PTK6-/- cells were transfected with empty vector (EV) or PTK6 cDNA then treated with either vehicle control or TNFα (100ng/ml) and IFNγ (500U/ml). Inlay shows successful overexpression of PTK6 cDNA vs. EV transfected cells. D) Either WT or PTK6-/- YAMCs were grown on transwell inserts at 2 X 10^5^ cells/ml until reaching 2-days post-confluence then treated with vehicle control (VC) or TNFα (100ng/ml) and IFNγ (500U/ml) for 24 hours. Permeability coefficients were calculated as described in methods. Error bars represent standard error (*p<0.05 compared to WT, n = 3). E) Westerns showing relative expression of PTK6 in siRNA treated and epithelial cells from PTK6-/- knockout mice (Please see S4 Fig for full blot scans).

Next, we sought to determine whether decreasing the expression of PTK6 was beneficial to barrier function in YAMC treated with TNFα/IFNγ cocktail. We first determined the impact of TNFα/IFNγ at various concentrations to identify the best concentration for the assay. Results showed that TNFα/IFNγ at 100ng/ml and 500U/ml yielded the strongest response (S1 Fig). Subsequently, electric cell-substrate impedance assays (ECIS) measuring the resistance of mock transfected vs. PTK6 siRNA transfected YAMC treated with TNFα/IFNγ cocktail showed improved resistance when PTK6 expression was decreased (Fig 2A, knockdown confirmation in Fig 2E). In addition, we were interested in determining whether PTK6 -/- cells showed improved barrier function in presence of inflammatory stimulation. Therefore, 2 X 10^5^ cells/ml of either PTK6-/- or WT YAMC cells were seeded on collagen I coated ECIS arrays then treated with vehicle control (0.1% BSA in PBS) or TNFα/IFNγ cocktail and measured resistance every 90 seconds. Values were normalized to timepoint zero. As shown in Fig 2B, WT YAMC cells showed approximately 40% drop in resistance upon treatment with TNFα/IFNγ, however PTK6-/- cells were insensitive to treatment. These results suggest that PTK6 participates in TNFα/IFNγ mediated epithelial barrier dysfunction.

In order to exclude the possibility that barrier changes observed in response to TNFα/IFNγ between PTK6 -/- and WT YAMC was erroneous, we transfected PTK6-/- cells with PTK6 cDNA or empty vector (EV) then compared TER values in response to treatment. Results showed that PTK6 expression in PTK6-/- cells sensitized monolayers to cytokine treatment and dropped resistance by approximately 10%, whereas PTK6-/- cells transfected with empty vector showed slightly enhanced resistance despite treatment (Fig 2C).

Next, to determine the differences in paracellular permeability between PTK6-/- and WT YAMC under inflammatory conditions, we utilized permeability assays for analysis of sodium fluorescein flux [25]. Either PTK6 -/- or WT YAMC cells (2 X 10^5^ cells/ml) were seeded on collagen I coated transwell inserts then grown to 2 days post-confluence. Fig 2D shows that the basal permeability of PTK6 -/- cells was approximately 40 fold less than that of WT cells and cytokine treated PTK6-/- cells were approximately 100 fold less permeable than that of WT cells (PTK6 expression knockout confirmed in Fig 2E). Importantly, permeability coefficients for treated vs. untreated PTK6-/- were not significantly different, however treatment of WT cells increased permeability by over 2 fold.

### The cytokine cocktail TNFα and IFNγ induced nuclear accumulation of FoxO1 was mediated by PTK6 in YAMC

**Fig 3:**
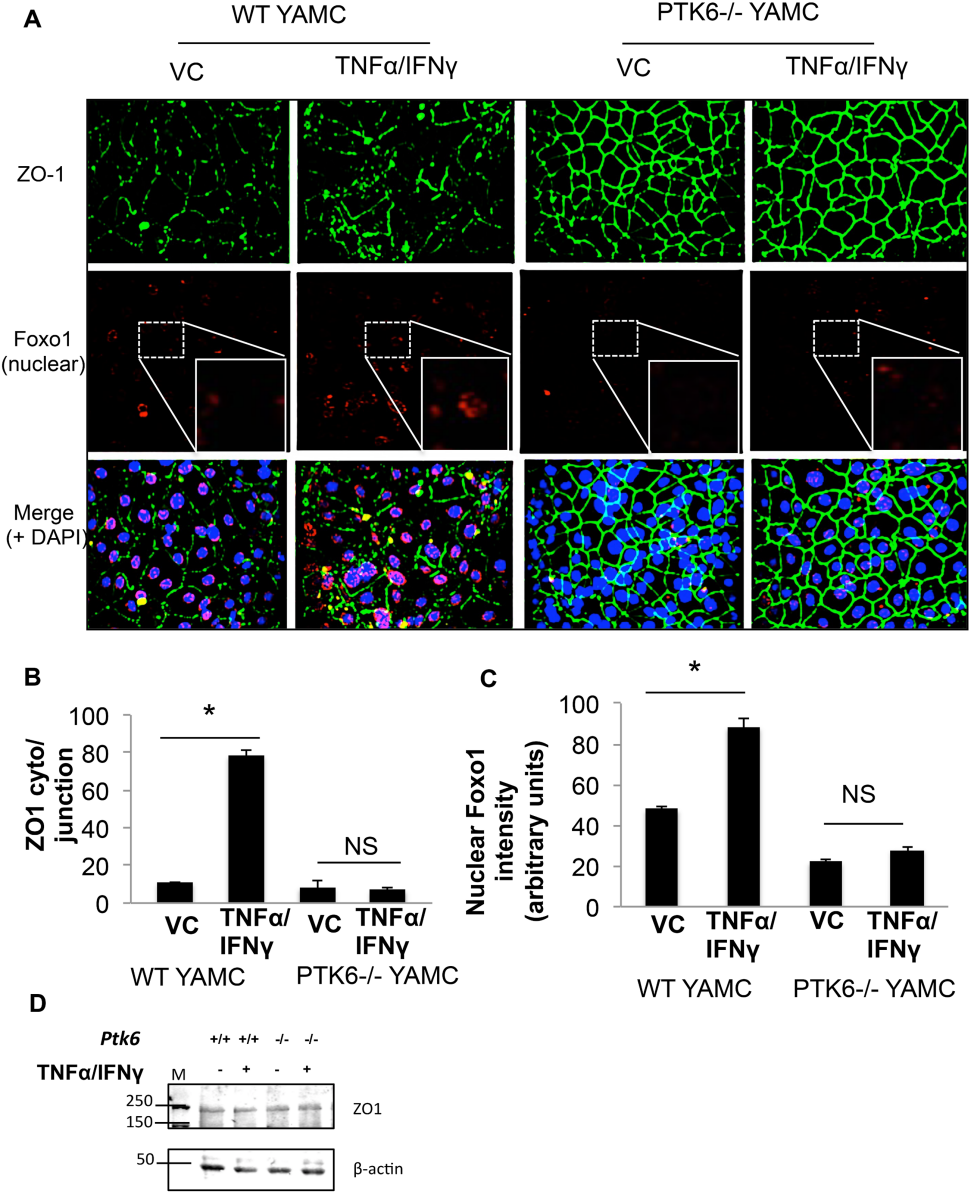
PTK6 contributes to the cytokine cocktail TNFα/IFNγ induced barrier dysfunction and FoxO1 nuclear translocation in YAMC. Post-confluent, mortalized WT or PTK6-/- YAMC monolayers were treated with vehicle control (0.1% BSA in PBS) or TNFα (100ng/ml) and IFNγ (500U/ml) 16 hours. A) Nuclei (blue), ZO-1 (green), and FoxO1 (red) were detected via confocal microscopy. B) Quantitation of the ratio of ZO-1 intensity in the cytoplasm over cell-cell junction from Fig 3A. C) Quantitation for nuclear FoxO1 intensity of FoxO1 stain in Fig 3A. D) Western blot indicating expression levels of ZO-1 in total cell lysates (please see S5 Fig for full blot scan). *p<0.05. Error bars represent SEM.

Next, considering that TNFα promotes FoxO1 nuclear accumulation [26] and ZO-1 reorganization which has been associated with impaired epithelial barrier function [27], and PTK6 knockout intestinal epithelial cells showed diminished FoxO1 nuclear accumulation [21], we hypothesized that PTK6 may mediate TNFα/IFNγ induced FoxO1 nuclear translocation and ZO-1 redistribution. Therefore, immunocytochemistry experiments were performed to visually identify nuclear accumulation of FoxO1 in PTK6-/- vs. WT YAMC after treatment with TNFα/IFNγ. As shown in Fig 3A and 3C, nuclear FoxO1 detection increased almost 2 fold upon TNFα/IFNγ treatment in WT YAMC cells, however no change was apparent in treated PTK6 -/- YAMC, suggesting that PTK6 may participate in a signaling mechanism that promotes the FoxO1 nuclear translocation resulting from TNFα/IFNγ treatment (quantitation in Fig 3C). Similarly, TNFα/IFNγ mediated cytosolic accumulation of the junction adapter protein ZO-1 was increased by approximately 4 fold in WT cells, but was unchanged in treated PTK6-/- cells (quantitation in Fig 3B) and ZO1 expression levels remained constant regardless of treatment or cell type (Fig 3D). These results suggest that the lack of response to TNFα/IFNγ in PTK6-/- cells may be the result of FoxO1 nuclear exclusion and/or retention of ZO-1 at the cell-cell junctions.

### Inhibition of PTK6 improved epithelial barrier integrity

**Fig 4:**
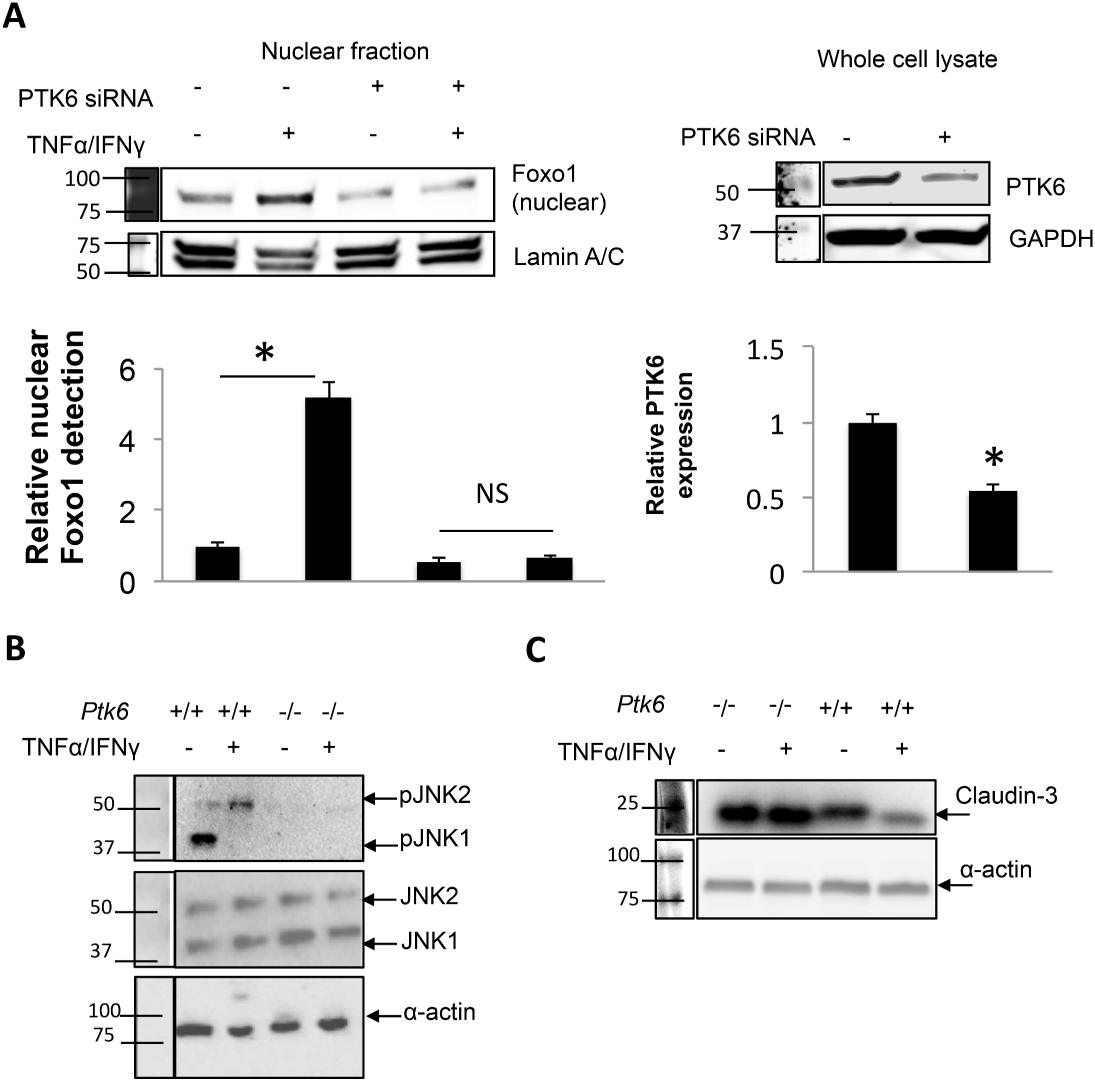
Inhibition of PTK6 prevents TNFα /IFNγ mediated FoxO1 nuclear accumulation, JNK activation, and improves claudin-3 expression. A) YAMC cultures were transfected with vehicle control or PTK6 siRNA then treated with vehicle control or TNFα/IFNγ for 16 hours. Nuclear fractions were harvested then assayed for presence of FoxO1. PTK6 was assayed in whole cell lysate to ensure knockdown. Densitometric calculations were based on 3 separate experiments (please see S4 Fig for full blot scan). p<0.05, n = 3. B, C) Either PTK6-/- or PTK6 +/+ were grown to confluence then treated with TNFα/IFNγ for 24 hours as indicated in Methods. Whole cell lysates were assayed by Western blot (please see S4 Fig for full blot scan). Results are representative of 3 separate experiments.

In order to identify the pathways PTK6 may regulate in order to increase permeability in response to inflammation, we treated cells expressing siRNA against PTK6 or PTK6 knockout cells and identified changes in markers of epithelial inflammation relative to the status of PTK6 expression. As shown in Fig 4A, inhibiting PTK6 expression significantly attenuated TNFα /IFNγ induced nuclear accumulation of FoxO1 by approximately 6 fold. Since JNK phosphorylation is known to increase FoxO1 nuclear accumulation, and we had identified JNK activation as being involved in TNFα /IFNγ mediated barrier dysfunction (Fig 1), we hypothesized that PTK6 may impact JNK activation. Indeed, ablation of PTK6 expression abolished JNK activation at basal or stimulated conditions (Fig 4B). Furthermore, since FoxO proteins act as repressors for certain claudin proteins, we probed claudin-1, claudin-2, claudin-3, and claudin-15 to determine whether PTK6 inhibition improved TNFα /IFNγ mediated downregulation of claudins. As shown in Fig 4C, PTK6 knockout prevented only claudin-3 downregulation (other claudins not shown) in response to TNFα /IFNγ, suggesting that PTK6 may play a role in JNK/FoxO1/Claudin-3 signaling.

### MicroRNA-93 targets PTK6

**Fig 5:**
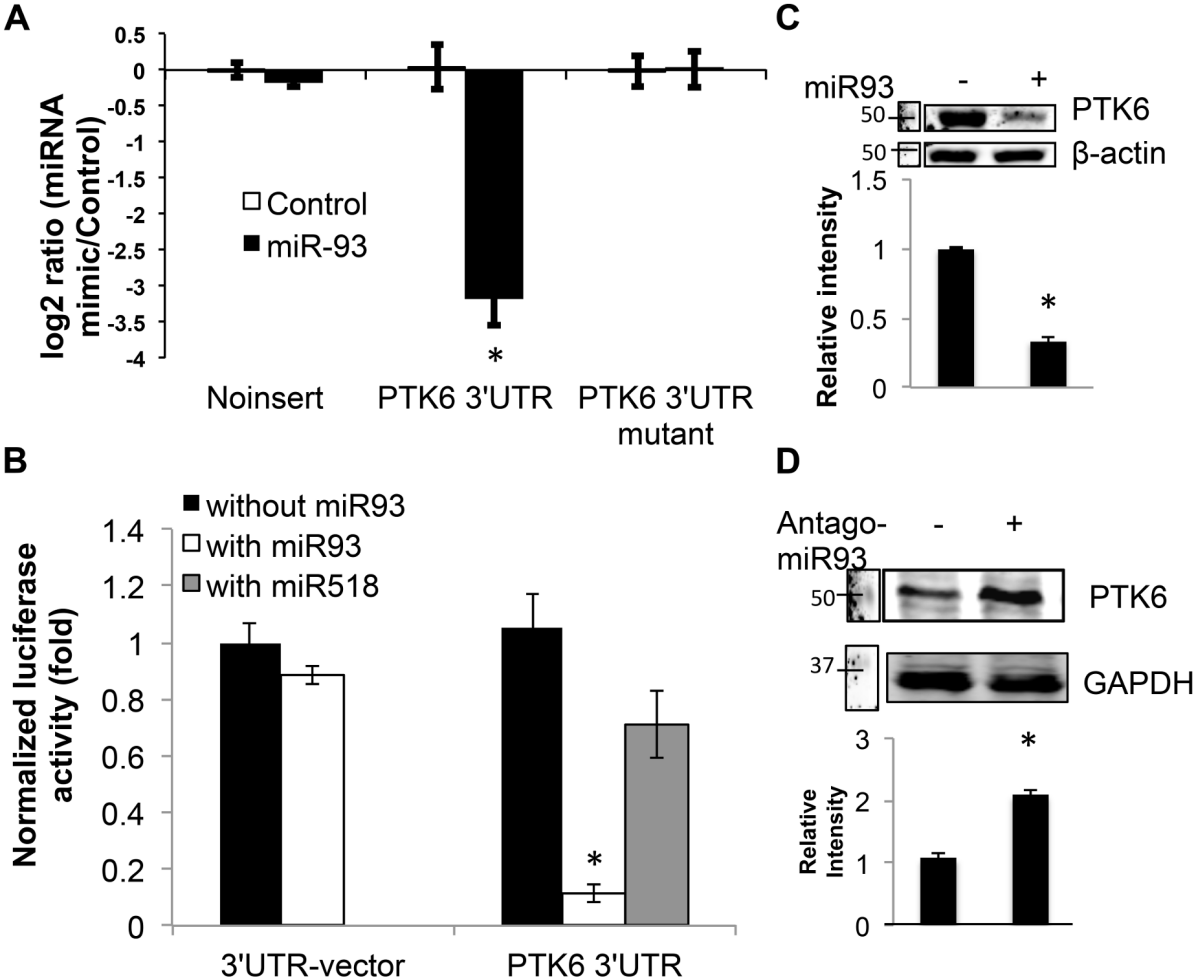
MicroRNA-93 is a confirmed epigenetic regulator of PTK6. A) YAMC were co-transfected with the indicated construct and scrambled sequence control or miR-93 then inhibition of luciferase excitation was assessed. B) YAMC were co-transfected with the indicated combinations of reporter/ oligo then luciferase excitation was assessed. C,D) YAMC were transfected with miR-93 mimic C) or miR-93 inhibitor D) then expression of PTK6 was determined by Western blot (please see S4 Fig for full blot scans). Results are representative of 3 unique experiments * p< 0.05.

Since microRNA often target multiple genes in a pathway in a manner that yields a physiological change, we sought to identify putative microRNA that target PTK6 and assess its efficacy in mitigating poor barrier function due to inflammation. Therefore, we utilized a number of sequence analysis suites to determine which miRNA may be appropriate to study. After considering 4 levels of criteria (site binding prediction, literature support, conservation of binding site, and number of available binding sites in the 3’UTR), miR-93 was identified as a candidate most worthy to pursue in improving barrier function and targeting PTK6 (S2 Fig). Subsequently, we investigated the targeting capacity of miR-93 in binding the 3’UTR of PTK6 using luciferase assays. As shown in Fig 5A, miR-93 inhibited luciferase activity by over 3 fold when preceded by the 3’UTR of PTK6, but not the luciferase reporter alone or when the two putative binding sites for miR-93 were mutated. In addition, miR-93 targeted the PTK6 3’UTR almost 3 fold more effectively than that of miR-518, another microRNA predicted to target PTK6 (Fig 5B). We then investigated whether miR-93 decreased PTK6 expression via Western blot. As shown in Fig 5C, miR-93 transfection reduced PTK6 by approximately 2-fold, and the inhibitor of miR-93 (antagomir-93) enhanced PTK6 expression by almost 2-fold (Fig 5D). These results indicate that miR-93 effectively targets PTK6.

### Intestinal epithelial cell treatment with microRNA targeting PTK6 (miR-93) improved cytokine induced barrier dysfunction

**Fig 6:**
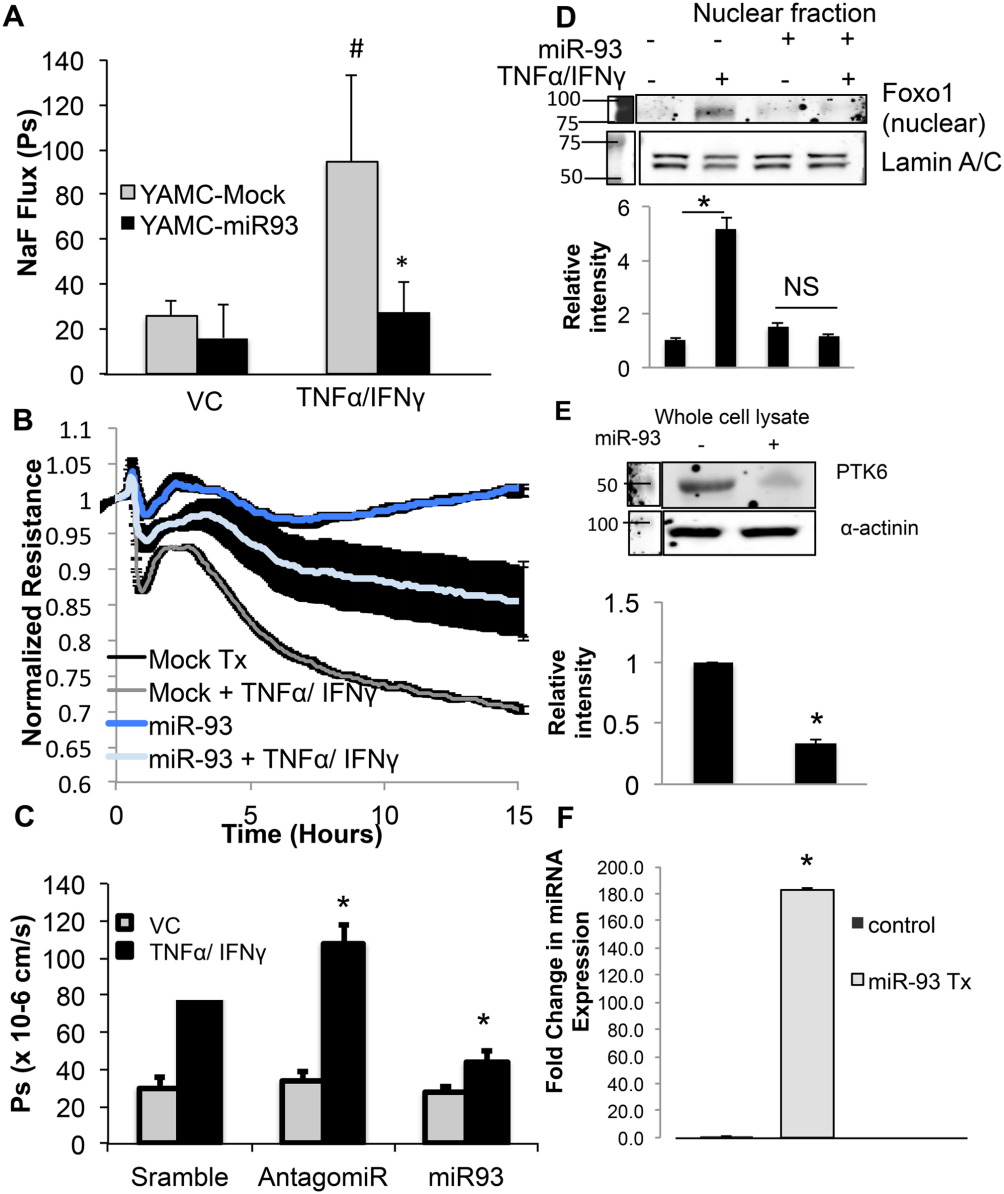
Intestinal epithelial cell treatment with microRNA targeting PTK6 (miR-93) improved cytokine induced barrier dysfunction. A) YAMC were electroporated with miR-93 or vehicle control (TE) and allowed to express for 48 hours. Monolayers were then treated with vehicle control or indicated cytokines for 24 hours then sodium fluorescein flux was assayed. Cell lysates were analyzed for PTK6 expression by Western blot. (*p<0.05 compared to treated mock, #p<0.05 compared to VC-mock. Error bars represent standard error, n = 4) C) YAMC were transfected as indicated then seeded on ECIS arrays at 2X10^5^ cells/ml followed by treatment with either vehicle control or TNFα/IFNγ. Resistance measurements were recorded every 90 seconds and values were normalized to timepoint zero. Shaded area represents standard error. C) YAMC were transfected with the indicated sequences for 48 hours then treated with vehicle control or TNFα/IFNγ as indicated in Methods. Flux to sodium fluorescine was measured by comparing fluorescence in luminal vs. abluminal compartments. D) YAMC cultures were transfected with vehicle control or mature miR-93 then treated with vehicle control or TNFα/IFNγ 16 hours. Nuclear fractions were harvested then assayed for presence of FoxO1. E) PTK6 was assayed in whole cell lysate to ensure adequate targeting of PTK6. F) qPCR results indicating successful transfection of miR-93 mimic in YAMC. Densitometric calculations were based on 3 separate experiments. (please see S5 Fig for full blot scans). *p<0.05, n = 3.

Since it was clear that PTK6 plays a significant role in inflammatory epithelial barrier dysfunction and miR-93 targets PTK6, we were interested in determining whether miR-93 treatment of YAMC improved cytokine mediated epithelial barrier dysfunction. To assess paracellular permeability, miR-93 transfected WT YAMC cells (2 X 10^5^ cells/ml) or mock transfected were seeded on collagen I coated transwell inserts then grown to 2 days post-confluence. Sodium fluorescein flux was measured in presence of either vehicle control or TNFα/IFNγ Results showed that miR-93 transfection of YAMC significantly attenuated TNFα/IFNγ mediated increase in sodium fluorescein flux while the cells transfected with mock displayed significant increase in permeability upon TNFα/IFNγ (Fig 6A). To corroborate these results, WT YAMC were electroporated with vehicle control (TE, mock) or miR-93 then seeded on ECIS arrays at 2 X 10^5^ cells/ml and allowed to express transfected sequences for 48 hours. Electroporated cells were then treated with vehicle control (0.1% BSA in PBS) or TNFα/IFNγ cocktail 16 hours with resistance measurements recorded every 90 seconds. Results showed that miR-93 transfected epithelial cells treated with TNFα/IFNγ showed improved resistance relative to treated mock transfected cells (Fig 6B). To confirm that the rescued permeability demonstrated by miR-93 was not due to tranfection with any nucleic acid, permeability assays were repeated with scrambled sequence, miR-93 inhibitor, or miR-93. As shown in Fig 6C, while the miR-93 antagomir exacerbated TNFα/IFNγ induced permeability by 20%, the miR-93 mimic attenuated permeability by several fold when compared to TNFα/IFNγ treated cells transfected with scrambled sequence. We then aimed to determine whether miR-93 attenuated FoxO1 nuclear accumulation despite TNFα/IFNγ treatment in a manner similar to PTK6 siRNA. Indeed, as shown in Fig 6D, miR-93 treatment prevented FoxO1 nuclear accumulation in a similar manner as targeting PTK6 with siRNA. These results suggest that miR-93 targeting of PTK6 may play a role in attenuating TNFα/IFNγ induced epithelial permeability.

## Discussion

Aberrant intestinal epithelial barrier function is a critical factor in several inflammatory diseases in the gut. Since PTK6 has been shown to play a role in basal epithelial barrier function, we sought to determine whether PTK6 also plays a role in mediating the response of inflammatory cytokines TNFα and IFNγ induced epithelial barrier dysfunction, and whether miR-93 targeting of PTK6 improved barrier function under such conditions. In this study, we provided evidence in support of 1) TNFα/ IFNγ induced expression and post-translational modification of PTK6; 2) PTK6-/- intestinal epithelial cells showed enhanced ZO-1 localization to cell-cell borders, and an attenuated response to TNFα/ IFNγ in ECIS and permeability assays; 3) Targeting PTK6 attenuated TNFα/ IFNγ driven JNK activation and claudin-3 downregulation 4) MiR-93 targeted PTK6 in YAMC and improved TNFα/ IFNγ mediated barrier dysfunction; and 5) FoxO1 nuclear accumulation resulting from TNFα/ IFNγ treatment was attenuated by PTK6 siRNA or miR-93.

The expression of several kinases are known to be upregulated by inflammatory cytokines including Src, fyn, and Yes [28]. Therefore, it is not surprising that PTK6 is also regulated in this manner. In fact, the PTK6 promoter contains two cis-acting elements with binding sites for NFκB and SP1, two transcription factors known to mediate gene regulatory activities of inflammatory cytokines such as TNFα and IFNγ in intestinal epithelial cells [29, 30]. Interestingly, it was found that PTK6-/- mice develop villi with augmented length and increased proliferation. This observation is consistent with previous findings that PTK6 is known to inhibit the activity of the serine/threonine kinase Akt in a manner that promotes FoxO1 accumulation in the nucleus [21]. Although we did not observe significant changes in Akt phosphorylation under these conditions, reports parallel to our results demonstrated that Foxo1 nuclear accumulation was robustly attenuated in PTK6-/- mice intestine [21].

After demonstrating that PTK6 mRNA was up-regulated by TNFα/ IFNγ, we then pursued the possibility that PTK6 may be involved in barrier dysfunction resulting from this treatment. By permeability and ECIS assays, we showed that diminishing PTK6 expression, by siRNA or knockout, improved barrier function in response to TNFα/ IFNγ. These findings were also observed in endothelial cells [14]. In addition, we showed that overexpressing PTK6 in PTK6-/- cells allowed TNFα/ IFNγ to impose barrier dysfunction when compared to cells overexpressing empty vector. Since the impact of targeting PTK6 contributed to sustained improvement in TNFα/ IFNγ barrier dysfunction, we further explored the impact of PTK6 expression status on signaling and changes in expression of genes that mediate prolonged epithelal barrier dysfunction, such as claudin-3, a tight junction protein that cricically maintains intestinal epithelial barrier integrity [31].

After the observation that claudin-3 downregulation was attenuated by targeting PTK6 expression, we investigated known signaling pathways to determine a possible mechanism that PTK6 potentiates barrier dysfunction. We and others have demonstrated that FoxO1 nuclear accumulation is a key factor in tight junction protein down-regulation [14],[32]. As a result, we assessed changes in FoxO1 nuclear accumulation with respect to TNFα/ IFNγ and PTK6 expression status and found that FoxO1 nuclear accumulation was attenuated in PTK6-/- cells. Although is generally accepted that decreased Akt activity impairs FoxO1 phosphorylation, an event that leads to FoxO1 exclusion from the nucleus [33], we found increased phosphorylation of FoxO1 and very little change in Akt phosphorylation in PTK6+/+ cells resulting from TNFα/ IFNγ treatment. This prompted us to explore another mechanism of FoxO1 nuclear accumulation, which is mediated by JNK activation [32]. Our results showing sustained JNK activation in response to TNFα/ IFNγ is consistent with other reports of increased JNK activity resulting from stress [34–36]. Our findings are in agreement with previous studies that showed nuclear translocation of FoxO1 was dependent on JNK activation, and this event was independent of Akt [35]. Furthermore, Corrozzino et al showed that JNK activation was a critical event in claudin protein down-regulation and subsequent epithelial barrier dysfunction [37]. Considering that our results show treatment of PTK6-/- showed decreased JNK activation, increased barrier function, diminished FoxO1 nuclear translocation and increased claudin-3 expression compared to PTK6+/+ cells, it seems plausible that PTK6 may be upstream of JNK, and JNK activation leads to FoxO1 nuclear translocation and subsequent repression of claudin-3 expression.

Since the therapeutic potential of microRNA is gaining momentum, we were interested in determining whether a microRNA targeting PTK6 may improve epithelial barrier function in the context of inflammation. Although it is widely known that microRNA have many targets, it is generally accepted that specific microRNA, or even microRNA families, target mRNA that encode proteins in the same pathway. Therefore, in demonstrating miR-93 supported FoxO1 exclusion from the nucleus, PTK6 down-regulation, and improved TNFα/IFNγ intestinal epithelial barrier dysfunction, we expect that this particular microRNA may be worth further exploration as a therapeutic agent with respect to intestinal epithelial permeability. In corroboration with our findings, a recent publication demonstrated that miR-93 activated PI3K/Akt signaling through direct suppression of PTEN [38]. This is consistent with the idea that microRNA often function to target a number of proteins that act together to tailor a particular response to stimuli. By targeting PTEN and enhancing Akt activity, the additional downregulation of PTK6 may impart a synergistic effect of miR-93 for FoxO1 nuclear exclusion through an ancillary mechanism involving JNK.

Overall, these results suggest that PTK6 is involved in inflammation driven intestinal epithelial barrier dysfunction, and miR-93 may serve as a novel intervention strategy to improve intestinal epithelial barrier function.

## Supporting Information

S1 Fig Peak changes in transepithelial resistance resulting from the indicated treatments on PTK6+/+ or PTK6-/- colonic epithelial cells. Monolayers of either cell type were grown on ECIS arrays as detailed in the Methods section. Peak decrease in normalized resistance following treatment is shown. Although 100ng/ml TNFα alone showed a similar response to TNFα/IFNγ (4^th^ column vs. last column), since the combination of TNFα/IFNγ more closely mimics that which is seen in vivo, we conducted subsequent experiments performed in this study with TNFα/IFNγ as stimulus.
(TIF)

S2 Fig Strategy for identifying miR-93 as potential microRNA that targets PTK6. A) First, predicted miRNA were identified using MirWalk “Gene-miRNA” prediction algorithm. This analysis produced 140 unique miRNA sequences. From this list, five microRNA known to be involved in inflammation or barrier function were identified for further analysis (Column 1). Probability scores on these five sequences were considered a “hit” when p<0.05 using MiRanda, PicTar, RNA22, and TargetScan (Column 2). Next, a pairwise alignment of the PTK6 3’UTR for human and mouse was conducted to determine areas of conservation (Column 3). The reverse complement (Column 4) of the seed sequence was searched for in the human (Column 5) or mouse PTK6 3’UTR (Column 6) (Ensemble) as well as in the pairwise alignment. The number of sites that were conserved across humans and mice are listed in Column 7. Two sequences showed binding potential in areas conserved between mice and humans. Mir-93 scored the highest in all categories. B) The MirWalk algorithm was used to predict 140 microRNAs that may bind the 3’UTR of PTK6. A literature search was conducted to determine significance of microRNAs predicted to target PTK6, five were taken for further analysis. Additional prediction algorithms were used to assess liklihood for binding (MiRanda, PicTar, RNA22, and TargetScan). Results of this analysis are shown in A. C) Pairwise alignment of the PTK6 3’UTR in humans and mice. The shaded areas represent alignment score for the indicated region. D) The 4 regions with matching sequences are shown, with the region corresponding to miR-93 shown in green.
(TIF)

S3 Fig Full western scans from Fig 1 blots. (TIF)

S4 Fig Full Western Blots for Figs 2E, 4A, 5C, 5D, 6D and 6E. Smaller blots are the result of cutting blot prior to antibody incubation for efficiency.
(TIF)

S5 Fig Whole Western blots for Figs 3D, 4B and 4C. (TIF)

## Abbreviations

PTK6: protein tyrosine kinase 6
YAMC: young adult mouse colonic epithelial cells
TER: transepithelial resistance
SH3: Src-homology 3 domain
WT: wild type
ECIS: electric cell-substrate impedance sensing
VC: vehicle control
