# Supplementary figures and images for: TNFα/IFNγ Mediated Intestinal Epithelial Barrier Dysfunction Is Attenuated by MicroRNA-93 Downregulation of PTK6 in Mouse Colonic Epithelial Cells
Source: PLoS One. 2016 Apr 27;11(4):e0154351. doi: 10.1371/journal.pone.0154351 (PMC4847919; doi:10.1371/journal.pone.0154351)

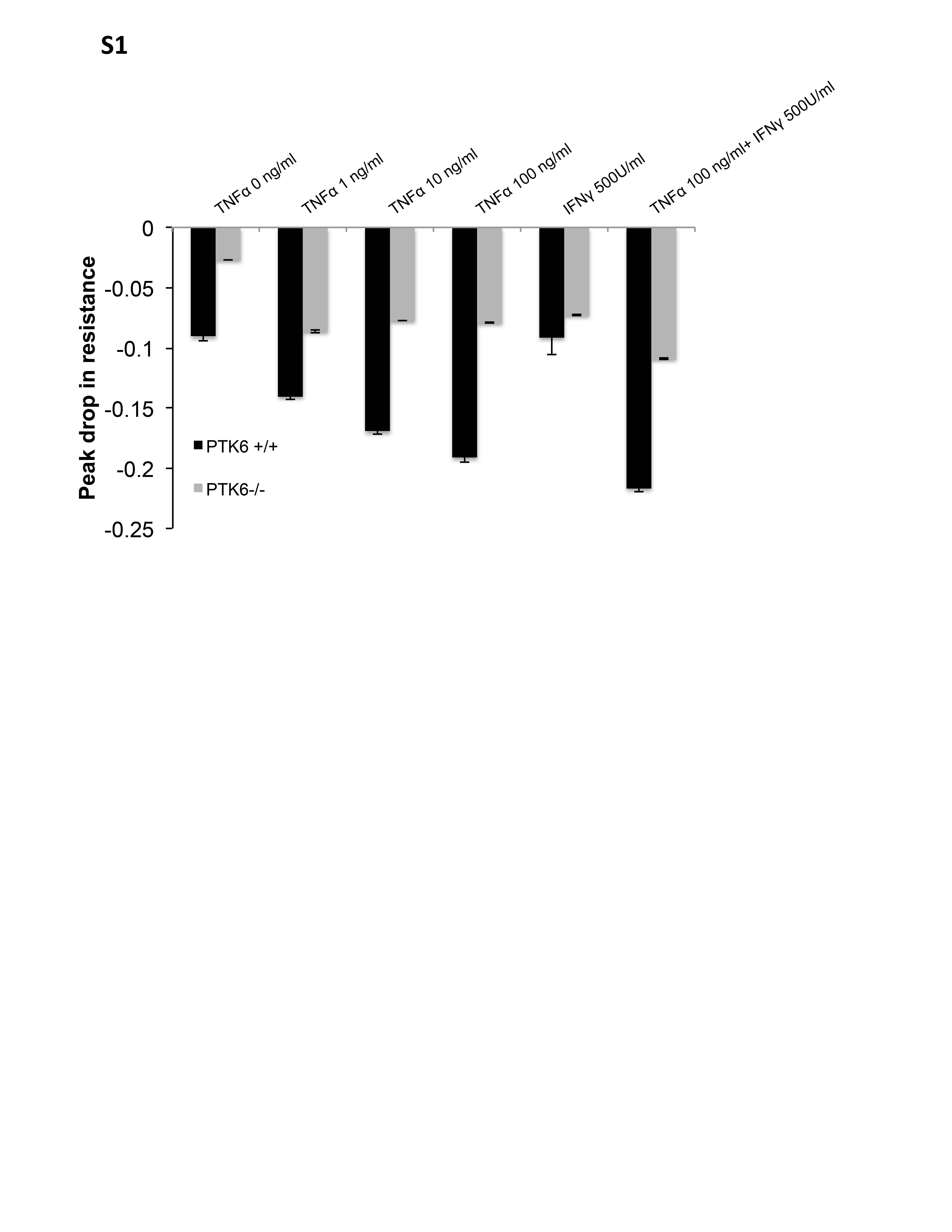

Supplement: S1 Fig — Monolayers of either cell type were grown on ECIS arrays as detailed in the Methods section. Peak decrease in normalized resistance following treatment is shown. Although 100ng/ml TNFα alone showed a similar response to TNFα/IFNγ (4th column vs. last column), since the combination of TNFα/IFNγ more closely mimics that which is seen in vivo, we conducted subsequent experiments performed in this study with TNFα/IFNγ as stimulus. (TIF) [file pone.0154351.s001.tif]

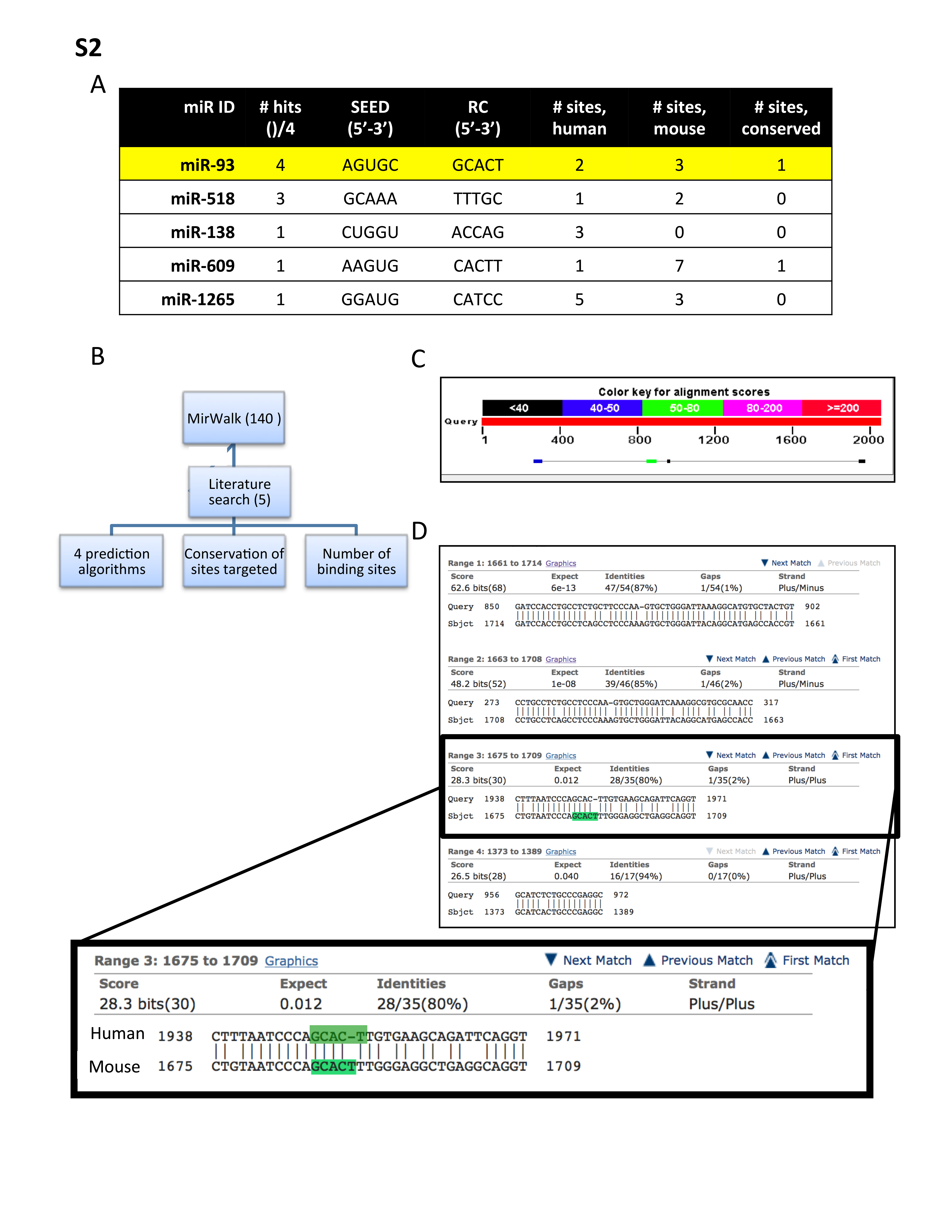

Supplement: S2 Fig — A) First, predicted miRNA were identified using MirWalk “Gene-miRNA” prediction algorithm. This analysis produced 140 unique miRNA sequences. From this list, five microRNA known to be involved in inflammation or barrier function were identified for further analysis (Column 1). Probability scores on these five sequences were considered a “hit” when p<0.05 using MiRanda, PicTar, RNA22, and TargetScan (Column 2). Next, a pairwise alignment of the PTK6 3’UTR for human and mouse was conducted to determine areas of conservation (Column 3). The reverse complement (Column 4) of the seed sequence was searched for in the human (Column 5) or mouse PTK6 3’UTR (Column 6) (Ensemble) as well as in the pairwise alignment. The number of sites that were conserved across humans and mice are listed in Column 7. Two sequences showed binding potential in areas conserved between mice and humans. Mir-93 scored the highest in all categories. B) The MirWalk algorithm was used to predict 140 microRNAs that may bind the 3’UTR of PTK6. A literature search was conducted to determine significance of microRNAs predicted to target PTK6, five were taken for further analysis. Additional prediction algorithms were used to assess liklihood for binding (MiRanda, PicTar, RNA22, and TargetScan). Results of this analysis are shown in A. C) Pairwise alignment of the PTK6 3’UTR in humans and mice. The shaded areas represent alignment score for the indicated region. D) The 4 regions with matching sequences are shown, with the region corresponding to miR-93 shown in green. (TIF) [file pone.0154351.s002.tif]

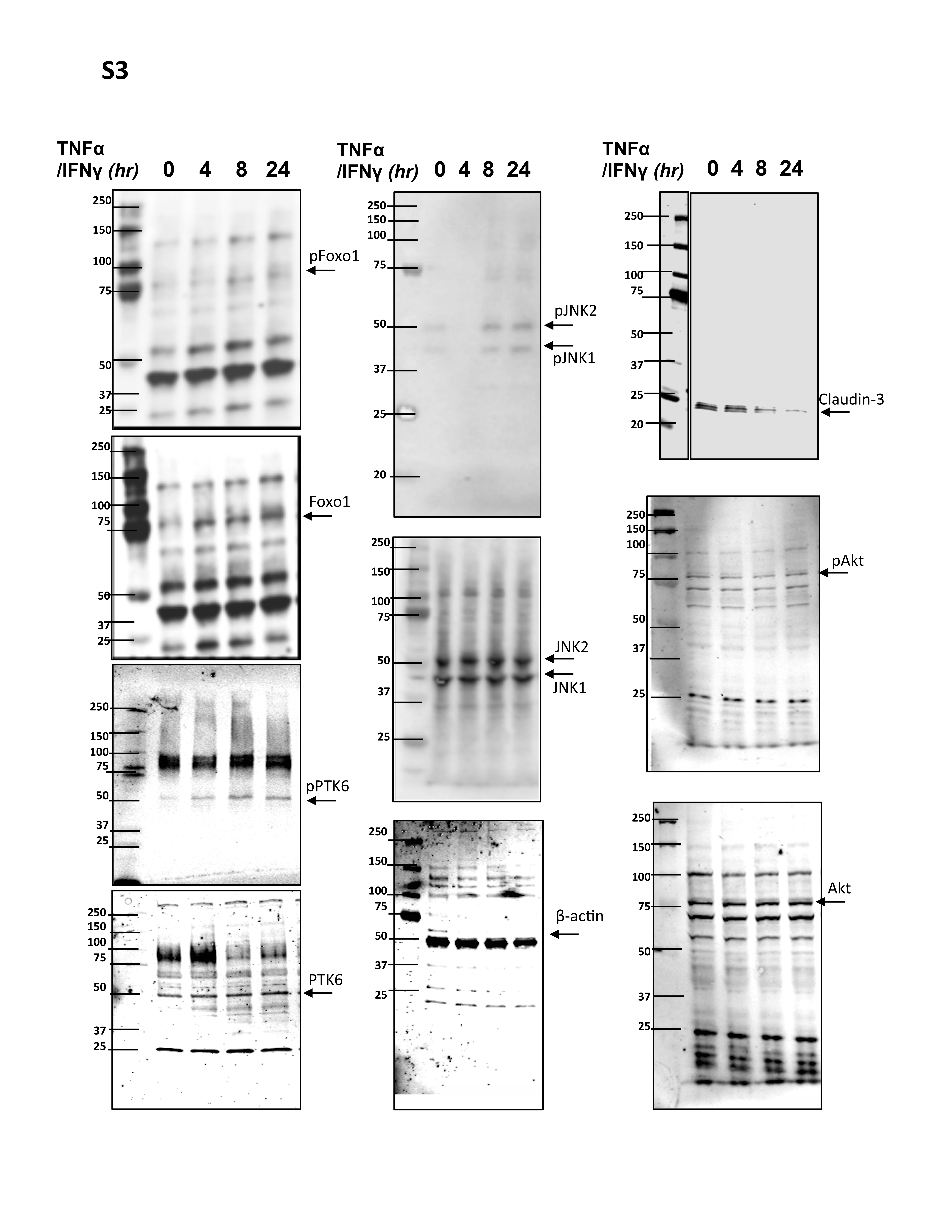

Supplement: S3 Fig — (TIF) [file pone.0154351.s003.tif]

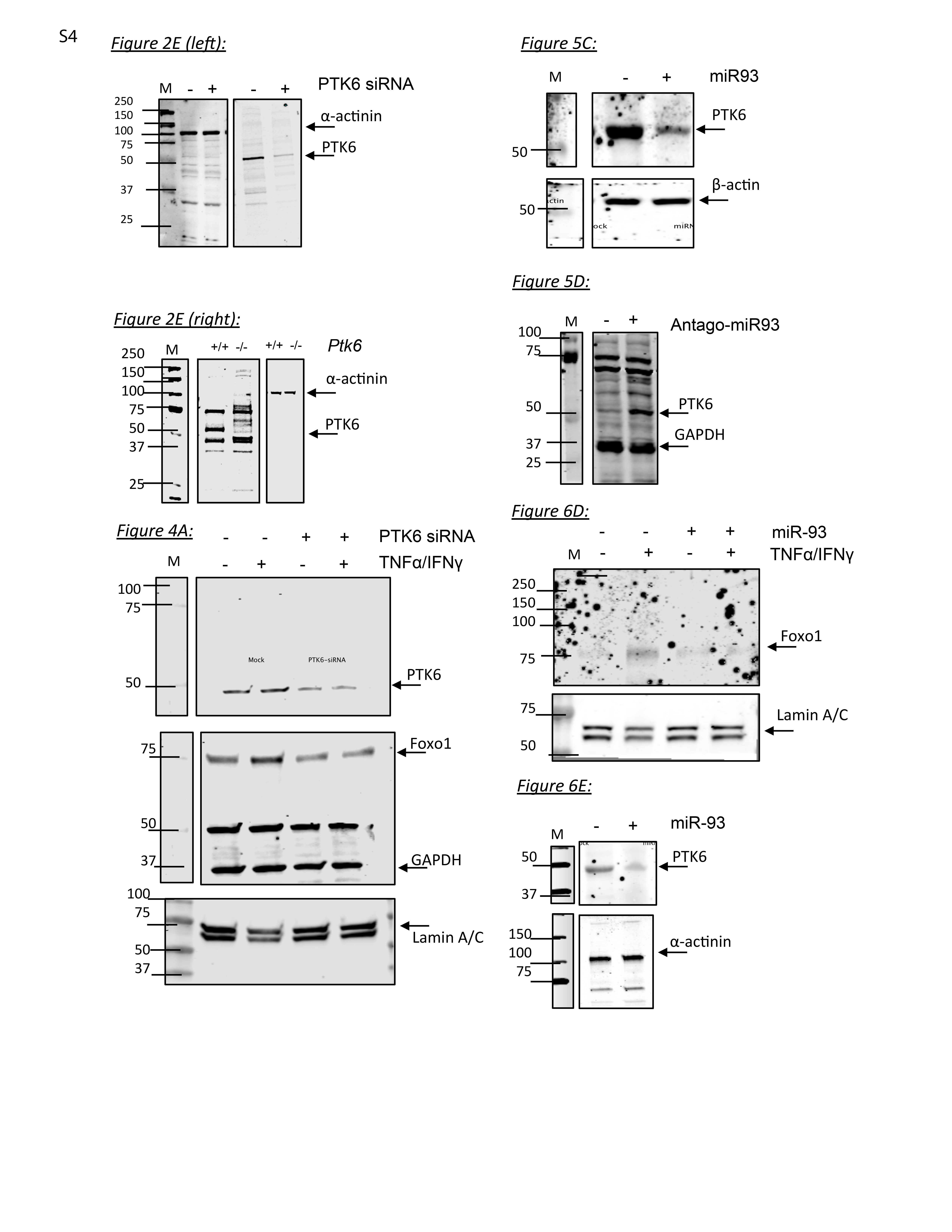

Supplement: S4 Fig — Smaller blots are the result of cutting blot prior to antibody incubation for efficiency. (TIF) [file pone.0154351.s004.tif]

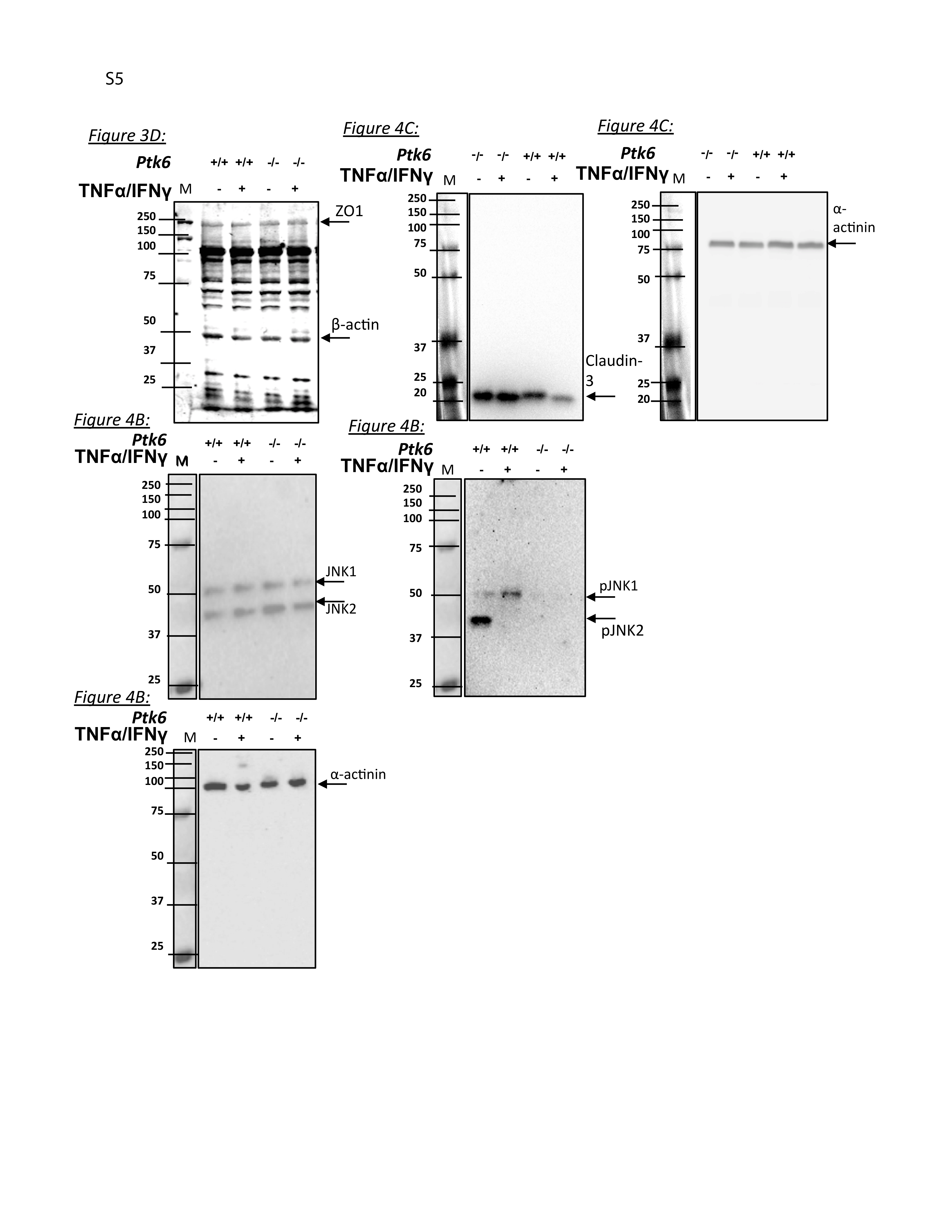

Supplement: S5 Fig — (TIF) [file pone.0154351.s005.tif]
